# Publication Rates of Abstracts Presented at the Urological Society of Australia and New Zealand Annual Scientific Meeting Keith Kirkland and Villis Marshall Session: A Decade‐Long Review

**DOI:** 10.1111/ans.70290

**Published:** 2025-08-13

**Authors:** Cheryl Fung, Lequang T. Vo, David Armany, Balasubramaniam Indrajit, Simon V. Bariol, Tania Hossack, Sriskanthan Baskaranathan, David Ende, Henry H. Woo

**Affiliations:** ^1^ Department of Urology Blacktown Mount Druitt Hospital Sydney Australia; ^2^ Department of Urology Dubbo Base Hospital Dubbo Australia

**Keywords:** abstract publication rate, KK/VM session, peer‐reviewed journals, urology research, urology SET trainees, USANZ ASM

## Abstract

**Background:**

The publication rate of abstracts at scientific meetings reflects research quality and impact, highlighting its dissemination within the academic and clinical communities. The Keith Kirkland and Villis Marshall (KK/VM) Session, held annually at the Urological Society of Australia and New Zealand (USANZ) Annual Scientific Meeting (ASM), features top abstracts from Urology SET Trainees. While the Board selects abstracts for presentation, their publication rates remain unclear. This study aims to determine the full‐article publication rate of abstracts presented at the KK/VM session from 2012 to 2022.

**Methods:**

The published Scientific Program of the USANZ ASM from 2012 to 2022 was retrospectively reviewed. All abstracts accepted for presentation at the KK/VM sessions were identified from the named plenary session. These abstracts were tracked for their subsequent publication status. Successful publication was defined as those identified in a search on PubMed using the abstract title and author name. Analysis of the successful publication was conducted.

**Results:**

One hundred and thirty‐five abstracts were identified. 51.85% were subsequently published in peer‐reviewed journals, most commonly in the *BJU International* (27.14%), *The Journal of Urology* (18.57%), and the *ANZ Journal of Surgery* (8.57%). The average time to publication was 14.58 months. The proportion of prospective studies among published (51.4%) and unpublished (52.3%) abstracts was similar.

**Conclusion:**

The USANZ ASM KK/VM session achieved a 51.85% full‐article publication rate from 2012 to 2022, surpassing the broader USANZ ASM rate. This underscores the high quality of research by Urology SET Trainees. Targeted support could boost publication rates, improving urology research dissemination.

## 1 Introduction

It is well recognized that most research abstracts presented at conferences will not progress to publication in peer reviewed journals. Even for prestigious conferences where there is significant competition and only a minority of abstracts are accepted for presentation, less than half of the presented abstracts will ultimately become a published manuscript.

The overall publication rate of abstracts presented at the Urological Society of Australia and New Zealand (USANZ) Annual Scientific Meeting (ASM) has previously been published. As part of the USANZ ASM scientific program, it hosts the Keith Kirkland and Villis Marshall (KK/VM) Session, showcasing the very best abstracts submitted by SET Urology Trainees.

The Board of Urology is the committee of the Royal Australasian College of Surgeons which, in conjunction with USANZ, carries responsibility for the training of urologists in Australia. Abstracts submitted by Urology SET trainees are assessed separately from all other abstracts and are reviewed by the Board of Urology. The highest ranked abstracts are selected for oral presentation in the KK/VM session, and those failing to be selected are then returned to the general abstract pool. Studies with endpoints that have the potential to have a direct clinical impact related to individual patient care are considered for the Keith Kirkland Prize. Studies where the primary outcomes are related to epidemiology or economic considerations, or associated with basic science or translational content, are considered for the Villis Marshall Prize.

Despite the prestige associated with acceptance for presentation at the KK/VM session, the subsequent progression of these abstracts to a full publication in peer‐reviewed journals remains uncertain. This study aims to quantify the publication rate of abstracts presented at the KK/VM session over a decade and to identify trends in their publication outcomes.

## 2 Methods

The published Scientific Program of the USANZ ASM from 2012 to 2022 was retrospectively reviewed. All abstracts accepted for presentation at the KK/VM sessions were readily identified from the named plenary session. These abstracts were tracked for subsequent publication in peer‐reviewed journals. A search using abstract titles and author names was performed via PubMed, MEDLINE, and Google Scholar. The consistency between the content of the presented abstract and that of the published article was confirmed. The articles were considered a match if they closely resembled the abstracts in terms of title, hypothesis, and study design. Data were analyzed to determine the publication rate and the journals of publication.

## 3 Results

A total of 135 abstracts were presented at the KK/VM session between 2012 and 2022. Of these, 70 abstracts (51.85%) were subsequently published in peer‐reviewed journals. There was variation in publication rates observed over 10 years, where it varied between 37.5% and 75%. This data are summarized in Table 1. Despite some annual variation, the publication rate was reasonably consistent over the 10‐year period, indicating stable dissemination of research findings from the KK/VM session. The most common journals for publication were *BJU International* (27.14%), *The Journal of Urology* (18.57%), and the *Australian and New Zealand Journal of Surgery* (8.57%).

**TABLE 1:** Rate of publication of abstracts presented at the KK/VM session of USANZ ASM from 2012 to 2022.

Year	No. of accepted abstracts	No. of published articles	Publication percentage	Site of publication
2012	15	10	66.66%	*Journal of Urology* (3), *ANZJS* (2), *BJUI*, *Urology*, other
2013	18	9	50%	*BJUI* (3), *ANZJS* (2), *JUS*, *Journal of Urology*, other
2014	16	6	37.50%	*Journal of Urology* (2), *BJUI*, other
2015	16	6	37.50%	*Journal of Urology* (2), *ANZJS*, *BJUI*, *World Journal of Urology*, other
2016	11	6	54.54%	*BJUI* (2), *Journal of Clinical Urology*, *Journal of Urology*, other
2017	12	6	50%	*European Urology*, *BMC Urology*, *Journal of Urology*, *BJUI*, other
2018	12	9	75%	*BJUI* (3), *BMC Urology*, *AJU*, other
2019	12	5	41.66%	*BJUI* (3), *International Journal of Urology*, *BAUS*
2020	Not formally presented at ASM due to COVID			
2021	12	7	58.33%	*BJUI* (3), *Journal of Urology* (2), *ANZJS*, *European Urology*
2022	11	6	54.54%	*Journal of Clinical Urology*, *Journal of Urology*, *European Urology*, *BJUI*, *BMC Urology*, other
Total	135	70	51.85%	*BJUI*: 19/70 (27.14%) *AUA*: 13/70 (18.57%) *ANZJS*: 6/70 (8.57%)

The average time to publication was 14.58 months, with wide variability: 13 studies were published within 1 month, 22 within 12 months, 12 within 24 months, and 11 took over 24 months. Notably, 12 studies were published prior to abstract presentation, suggesting either overlapping timelines or delayed abstract submission processes.

Our analysis of 70 published articles revealed that clinical research predominated, with prospective studies representing the majority (*n* = 36, 51.4%), followed by retrospective studies (*n* = 27, 38.6%). Preclinical research accounted for a smaller proportion, including one animal study (1.4%) and six in vitro/ex vivo studies (8.6%). Among the 65 non‐published abstracts, the distribution mirrored published studies in some respects: 34 were prospective (52.3%), 27 retrospective (41.5%), and 4 qualitative (6.2%). Notably, while prospective studies had the highest absolute numbers in both published and non‐published cohorts, their publication rate (51.4% of published vs. 52.3% of non‐published) suggests that study design alone did not guarantee publication success. The absence of published qualitative studies (despite four abstracts) may reflect challenges in translating such work into journal articles, though the small sample size limits definitive conclusions.

## 4 Discussion

The findings of this study demonstrate that more than half of the abstracts presented at the KK/VM session progress to publication in reputable peer‐reviewed journals. This publication rate surpasses the overall rate reported for the USANZ ASM (30%) [1]. Publication rates for national meetings are not dissimilar to that of the USANZ ASM. In particular, the overall publication rates of abstracts presented at the Canadian Urological Association ASM and the British Association of Urological Surgeons ASC were 45.4% and 42%, respectively [2, 3].

In contrast, publication rates for multidisciplinary surgical meetings can be lower, such as the Royal Australasian College of Surgeons Annual Scientific Congress (RACS ASC) which demonstrated a publication rate of 26% from 2010 to 2014 [4]. It is notable that Urology is one of the few specialties that have not regularly participated at the RACS ASC. The consistently high publication rate over the evaluation period suggests a sustained quality of research presented at the KK/VM session, which aligns with rates reported for prestigious major international conferences such as the American Urological Association Annual Meeting (51.9%) and the European Association of Urology Congress (47.35) [5, 6].

Our study focused exclusively on abstracts from the KK/VM prize sessions and did not evaluate publication rates for other USANZ ASM awards, including BAUS Trophy, Platinum Trophy, Best Oncology Presentation, Young Investigator Award, Low‐Arnold Award in Female and Functional Urology, or Alban Gee Award. To date, there has been no study done to review publication rates of abstracts accepted for these prizes; therefore, comparison is not available. Expanding the analysis to include these prizes would be valuable for identifying differences in publication outcomes across award types. This could be a direction for future research, particularly to determine whether certain prize categories correlate with higher academic impact.

Despite the relatively high publication rate, there remains potential for improvement. Achieving close to a 100% publication rate is an attainable goal with enhanced support and guidance for trainees in project design, manuscript preparation, and submission processes. We believe publication rates could be further improved through targeted interventions such as follows: (1) Mentorship programs: pairing early‐career researchers with senior authors to refine manuscripts post‐presentation. (2) Pre‐submission peer review: offering structured feedback within institutions or societies prior to journal submission. (3) Incentivizing timely publication: recognizing published abstracts in subsequent ASM programs to encourage follow‐through. (4) Journal partnerships: collaborating with urology journals to fast‐track high‐quality abstracts from prize sessions.

Dickersin et al. in their paper found “lack of time” to prepare a manuscript as the main reason cited by investigators for not publishing their abstract [7]. Arora et al. proposed several factors may explain why conference presentations do not always result in journal publications. First, research that is compelling enough for conference presentation may not necessarily meet the more rigorous methodological standards required for journal publication. Additionally, publication outcomes are influenced by author intent—some presenters submit abstracts primarily to fulfill training requirements or specialty application criteria rather than with the intention of pursuing full publication. For instance, trainees may present work as part of their program obligations without plans to develop it into a manuscript. These considerations suggest that non‐publication may reflect differences in author priorities rather than the inherent quality of the research itself [8].

## 5 Limitations

Our retrospective design relied solely on published conference programs, which lacked critical details such as prize status (winners vs. non‐winners), presenter characteristics (SET level, gender, institution), and distinction between VM and KK presentations. This prevented analysis of potential demographic or prize‐related publication biases.

Our study focused exclusively on KK/VM presentations, excluding other USANZ prizes (BAUS Trophy, Low‐Arnold Award, etc.). There is also a lack of investigation into the reasons behind the non‐publication of abstracts and the omission of analysis regarding the impact factors of publications, such as research methodology, sample size, established funding sources, and whether the studies were multicentric. Exploring the reasons for non‐publication through qualitative interviews with presenters would provide valuable context; however, such an analysis was beyond the scope of our current study, which was designed as a retrospective review of published abstracts.

Publication identification depended on abstract‐to‐article matching, which may have missed the articles published in non‐indexed journals and studies with significant title/author changes. Our study may not have captured publications that remain unpublished, particularly those related to the most recent years' abstracts.

Future research could build on our findings by incorporating mixed‐methods approaches, including surveys or interviews with authors of unpublished abstracts. This would help identify systemic or individual obstacles to publication and inform strategies to improve translational rates from presentation to peer‐reviewed literature.

Despite these limitations, our study provides the first comprehensive analysis of KK/VM publication outcomes using systematic methodology. The large sample size (70 publications) and decade‐long timeframe offer meaningful insights into publication patterns. Future prospective studies could address these gaps by incorporating prize status tracking and author surveys.

## 6 Conclusion

The KK/VM session at the USANZ ASM has demonstrated a notable publication rate of 51.85% for abstracts presented between 2012 and 2022. This rate exceeds that of the broader USANZ ASM and highlights the strong calibre of research conducted by SET Urology Trainees. With targeted support, the goal of achieving an even higher publication rate is feasible, fostering greater dissemination of high‐quality urological research.

## Disclosure

The authors have nothing to report.

## Data Availability

The data that support the findings of this study are available on request from the corresponding author. The data are not publicly available due to privacy or ethical restrictions.
